# Carbon nanotube incorporation in PMMA to prevent microbial adhesion

**DOI:** 10.1038/s41598-019-41381-0

**Published:** 2019-03-20

**Authors:** Kyoung-Im Kim, Dong-Ae Kim, Kapil D. Patel, Ueon Sang Shin, Hae-Won Kim, Jung-Hwan Lee, Hae-Hyoung Lee

**Affiliations:** 1 Department of Biomaterials Science, School of Dentistry, Dankook University, Cheonan, 31116 South Korea; 2 Institute of Tissue Regeneration Engineering (ITREN), Dankook University, Cheonan, 31116 South Korea; 3 Department of Dental Hygiene, Kyungwoon University, Gumi-si, South Korea; 4 Department of Nanobiomedical Science & BK21 PLUS NBM Global Research Center for Regenerative Medicine Research Center, Dankook University, Cheonan, 31116 South Korea; 5 UCL Eastman-Korea Dental Medicine Innovation Centre, Dankook University, Cheonan, 31116 Republic of Korea

## Abstract

Although PMMA-based biomaterials are widely used in clinics, a major hurdle, namely, their poor antimicrobial (i.e., adhesion) properties, remains and can accelerate infections. In this study, carboxylated multiwalled carbon nanotubes (CNTs) were incorporated into poly(methyl methacrylate) (PMMA) to achieve drug-free antimicrobial adhesion properties. After characterizing the mechanical/surface properties, the anti-adhesive effects against 3 different oral microbial species (*Staphylococcus aureus*, *Streptococcus mutans*, and *Candida albicans*) were determined for roughened and highly polished surfaces using metabolic activity assays and staining for recognizing adherent cells. Carboxylated multiwalled CNTs were fabricated and incorporated into PMMA. Total fracture work was enhanced for composites containing 1 and 2% CNTs, while other mechanical properties were gradually compromised with the increase in the amount of CNTs incorporated. However, the surface roughness and water contact angle increased with increasing CNT incorporation. Significant anti-adhesive effects (35~95%) against 3 different oral microbial species without cytotoxicity to oral keratinocytes were observed for the 1% CNT group compared to the PMMA control group, which was confirmed by microorganism staining. The anti-adhesive mechanism was revealed as a disconnection of sequential microbe chains. The drug-free antimicrobial adhesion properties observed in the CNT-PMMA composite suggest the potential utility of CNT composites as future antimicrobial biomaterials for preventing microbial-induced complications in clinical settings (i.e., Candidiasis).

## Introduction

For almost three quarters of a century, poly(methyl methacrylate) (PMMA) has been a clinically accepted biomaterial in the medical/dental fields, where it is used in various types of implantable or removable devices (e.g., denture, cement, facial prostheses, and temporary tooth restorative materials) due to its desirable biological/mechanical properties, easy production, and relatively low cost^1–3^. However, its limited antimicrobial properties remain a major hurdle to overcome because of the possibility of biomaterial-derived infections when in contact with body or when implanted in tissue for tissue regeneration or anatomical restoration^4,5^. One of the most promising approaches that has been utilized recently to tackle this drawback is the incorporation of nanosized biomaterials^6–9^

Silica-based nanoparticles such as mesoporous silica nanoparticles (MSN) and calcium-doped silica nanoparticles, as well as metal-based nanoparticles (e.g., gold nanoparticles and titanium nanoparticles), have been explored with their intrinsic advantages as additives in biomaterials: high suspension stability, therapeutic activities (e.g., preventing microbial adhesion), mechanical reinforcement properties, biomolecule (i.e., drug or ion) loading/releasing capacities, and excellent biocompatibility^10–16^. Recently, MSNs were conjugated with PMMA as a nanoadditive, and drug-free antimicrobial adhesion properties (20~30% reduction compared to the MSN-free control) were achieved when more than 2.5 wt% of MSN was incorporated, which was primarily due to the enhanced generation of a hydration layer^17^. However, these anti-adhesive effects were still suboptimal. To tackle these low anti-adhesive effects, antimicrobial drugs (50~90%, i.e., amphotericin B or silver sulfadiazine) were also loaded into the MSN-incorporated PMMA, but the complexity of loading/recharging drugs in nanoparticle-PMMA complexes remains a major hurdle to the future clinical use of these materials^17–19^. Therefore, to obtain PMMA with drug-free antimicrobial adhesion properties, new trials are needed^20,21^.

As candidates, carbon-based nanomaterials such as graphene oxide nanosheets (nGOs), carbon nanotubes (CNTs), and fullerene have been highlighted due to their innate antimicrobial effects when directly contacted with microbes^22–26^. Moreover, the antimicrobial (i.e., adhesion-preventing) effects of these nanomaterials, which are caused by the generation of a hydration layer on the biomaterial surface or direct interaction with microbial membranes, remained when functionalized nGOs or CNTs were incorporated into a bulk biomaterial^27,28^. Weak intermolecular adhesion forces between microorganisms and CNTs, direct cell membrane perturbation and elevated oxidative stress via electronic control were suggested as possible mechanisms of CNT or their composites^26,29^. Along with the many merits of incorporating CNTs into PMMA, such as enhanced mechanical properties and increases in volumetric stability during polymerization, CNT-PMMA complexes have been examined for their clinically applicable drug-free antimicrobial adhesion properties^30,31^

In this study, we focused on incorporating CNTs that had been carboxylated (-COOH), which increases their dispersibility in solution, into PMMA to induce effects that prevent microbial adhesion in the absence of any antimicrobial drugs. After investigating the morphology and surface characteristics of CNT-incorporated biopolymer (CNT-PMMA) (containing 0.25 to 2 wt% CNT), the resulting mechanical properties and anti-adhesive effects against 3 different representative microbes related to oral environment-induced infection were scrutinized. Furthermore, highly magnified scanning electron microscopy (SEM) images and images of microbes were captured to reveal morphological changes induced by the anti-adhesive properties of CNT-PMMA. This work provides information relevant to the utilization of nanoparticles (e.g., CNTs) for the future development of clinically applicable drug-free biomaterials (e.g., PMMA) that can prevent microbial adhesion.

## Material and Methods

### Characterization of CNT-incorporated PMMA

Pristine CNTs with a 15–20 nm outer diameter and 10–20 μm length were purchased from EMP (EM-Power Co., LTD, Korea) and were functionalized to have carboxyl groups for enhanced dispersibility according to a previously reported acid oxidation methodology^32^. Briefly, pristine CNTs were added to a H_2_SO_4_/HNO_3_ aqueous solution (1:1 vol%), refluxed at 80 °C for 2 days, washed, filtered with a 0.4 µm Millipore polycarbonate filter membrane, and dried. After carboxylation, CNTs were characterized by transmission electron microscopy (TEM; JEOL 7100, Peabody, MA, USA). CNTs were conjugated with chemically polymerized PMMA products (Orthocryl resin, Dentaurum, Ispringen, Germany) to avoid any damage to the CNTs during heat polymerization. Composites were prepared with CNTs at concentrations of 0.25, 0.5, 1.0 and 2.0% by weight relative to PMMA powder by homogeneously dispersing CNTs in liquid methyl methacrylate (MMA) monomer under sonication. The homogeneous dispersion of CNTs in monomer solution within 40 minutes, which was the maximum time required for fabricating CNT composites, was confirmed by a Turbiscan analyzer (LAB Science). The Turbiscan analyzer scanned the turbidity change upon aggregation of particles from the middle of a cuvette (h = 40 mm) via the transmittance (%) at certain intervals^33^. A significant change in transmittance over time indicates inhomogeneous suspension or aggregation formation over time^34^. After replacing a predetermined amount of PMMA powder with equivalent amounts of CNTs (0.25~2.0%), the PMMA powder and CNT-monomer mixture were manually mixed at a PMMA(-CNT) powder (g) to monomer solution (ml) ratio of 2:1. The powder (g) and liquid (ml) ratio (2:1) was based on the manufacturer’s recommendation. The mixture was poured into silicone molds with various dimensions to prepare mechanical and antibacterial specimens. After polymerization at 40 °C for 20 minutes under 3 bars of pressure using a MultiCure machine (Vertex, Zeist, Netherlands), disks (d = 11.5 mm and h = 1.5 mm) were sectioned, polished with up to 220 or 2400 grit SiC paper, and used for investigations. This high polished disk was used for experiments unless otherwise mentioned. To make rough surfaces that mimic scratched denture surfaces while in use, only 220 grit SiC paper was used for polishing. Specimens fully polished with up to 2400 grit SiC paper were used to represent freshly polished surfaces, i.e., the surfaces when PMMA-based devices are delivered to patients. All disks used for antibacterial tests and cytotoxicity tests were exposed to ethylene oxide (EO) gas for sterilization^35^. Fourier transform infrared spectroscopy with attenuated total reflectance (FTIR-ATR; Varian 640-IR, Australia) was used for investigating the prepared CNT-incorporated PMMA samples in the range of 4000–400 cm^−1^ referred to a previously reported method^17,34^. The surface morphology and roughness was examined by SEM (Sigma 500, ZEISS, Jena, Germany) and surface profiler (n = 10, Ra, SJ-400, Mitutoyo, Japan) respectively, and contact angle with distilled water (DW) were measured by a Phoenix 300 system (n = 5, SEO, Suwonsi, Gyunggido, Korea) as described in detail elsewhere^17,36^.

### Mechanical properties

Bar-shaped specimens (65 × 10 × 3.3 mm), polished with up to 1200 grit SiC paper, were prepared. After they were immersed for 48 h at 37 °C in DW, they were placed in an Instron 5966 machine (500 N load cell, Norwood, MA, USA) equipped with a DW chamber at 37 °C for 3-point flexural tests with a span length of 14 mm according to the ISO standard (ISO 20795-2)^37^. The ultimate flexural strength and elastic modulus were measured at a crosshead speed of 5 mm/min (n = 8)^38,39^. Fracture toughness (n = 8) was tested using specimens (30 × 6.5 × 3.3 mm) with a notch (2.7 mm deep and 0.5 mm wide) according to the ISO standard (20795-2) with a span length of 24 mm^37^. Briefly, specimens were polished with up to 1200 grit SiC paper, incubated in DW for 7 days at 37 °C, dried at room temperature (RT), and positioned in the abovementioned Instron machine to measure the maximum strength at fracture. Specimens for toughness testing (n = 8) were incubated in DW for 7 days at (37 ± 1) °C and conditioned at 23 ± 1 °C for 60 ± 15 minutes prior to testing. The total fracture work (J/m^2^) was calculated from the integral of the load-displacement curve (J) during the fracture toughness test using Bluehill 2 software (Instron). Charpy impact tests (n = 8) were performed on bar-shaped specimens (50 × 6 × 4 mm) with V-shaped notches (1.2 mm deep, ISO 179 type-A notch) after the specimens had been polished with up to 1200 grit SiC paper, incubated in DW for 7 days at 37 ± 1 °C, and conditioned at 23 ± 1 °C for 60 ± 15 minutes. These tests were conducted according to the ISO standard using a testing device (HIT5.5 P, Zwick, Ulm, Germany) with a 0.5 J Charpy pendulum^40^.

### Antimicrobial study

The prepared disk specimens (∅ = 11.5 mm and d = 1.5 mm) were polished with SiC papers up to 220 or 2400 grit and exposed to EO gas for sterilization^35^. *Staphylococcus aureus* (*S. aureus*, ATCC 6583), *Streptococcus mutans* (*S. mutans*, ATCC 25175) and *Candida albicans* (*C. albicans*, ATCC 10231) were cultured as stated by the manufacturer’s protocols^19^. Briefly, *S. aureus, S. mutans*. and *C. albicans* were cultured aerobically in trypticase soy broth (BD, Franklin Lakes, NJ, USA), brain heart infusion broth (BD), yeast mold broth (BD), respectively, at 37 °C with a humidified incubator (Panasonic, Osaka, Japan). Subculturing was performed every other day. After determining the number of colony forming units on agar-based solid media, a 100 µl solution (10^6^ microbes) consisting of a microbial suspension (10 µl, with 10^8^ microbes/ml) of each species in the log phase of growth and artificial saliva (90 µl)^41^ was initially seeded on the surface of a disk. After 1 h of incubation at 37 °C in a 5% CO_2_ incubator to allow microbes to adhere to the disks, two washes with phosphate buffered solution (PBS, pH 7.4 Sigma) were used to remove nonadherent microbes. After washing, the disks were moved to new culture plates and further incubated with 10% PrestoBlue (Molecular Probes, USA) in 1 ml of microbial culture media for the determined times, and the optical density (OD; 570 nm–600 nm) of 100 µl of media was measured at each time point (0~4 h) using a 96-well microplate reader (BioTek, Winooski, VT, USA). The optical density was normalized to the value from the positive control (CNT 0%, n = 5) at the maximum incubation time (3 or 4 h) until saturation occurred in the culture plate control (experimental control). The microbial adhesion of each specimen (CNT 0, 0.25, 5, and 1%) was calculated as % relative to CNT 0% after 3 or 4 h of incubation depending on the type of microorganism. In addition, to check the numbers and morphology of adherent *C. albicans*, FUN-1 staining (Thermo Fisher Scientific, Waltham, MA, USA) was performed according to a previously described methodology^19^. Briefly, a solution of FUN-1 at a final concentration of 20 µM was added and incubated at 37 °C in the dark for 30 minutes. Then, images were obtained by confocal microscopy (LSM 510, Zeiss, Switzerland). In this system, green fluorescence accumulates throughout the cytoplasm, indicating the presence of *C. albicans* cells and their morphology. The procedure about FUN-1 staining is described in detail elsewhere^42–44^.

### Cytotoxicity testing

Immortalized human gingival oral keratinocytes (IHOKs) were chosen to represent the cell type of the outermost oral mucosal layer, which is composed of keratinocytes in humans^45,46^. After IHOKs (n = 6, 1 × 10^4^ cells) were seeded in each well of 96-well plates and incubated for 24 h at 37 °C in a humidified atmosphere with 5% CO_2_, extracts from specimens at a specimen surface-solution (DW) ratio of 3 cm^2^/mL were prepared at 37 °C in a humidified atmosphere with 5% CO_2_ under shaking conditions (180 rpm) according to the ISO standard^47^. Then, the original extract was mixed with 2X DMEM/F-12 (3:1) supplemented with 2% penicillin/streptomycin (Invitrogen, Waltham, MA, USA) and 20% fetal bovine serum (Gibco) at a 1:1 ratio (volume) to make 50% extract. Serial dilutions using 1X DMEM/F-12 (3:1) supplemented with 1% penicillin/streptomycin (Invitrogen, Waltham, MA, USA) and 10% fetal bovine serum (Gibco) were performed to obtain 25, 12.5, and 6.25% extracts. Each extract (50, 25, 12.5, and 6.25%) was added to each well of the 96-well plates containing adherent IHOKs and cocultured for another 24 h at 37 °C in a humidified atmosphere with 5% CO_2_. After 24 h of culturing, cytotoxicity was measured using a WST assay according to previously described methods (OD 450 nm)^48,49^.

### Statistical analysis

The data are shown as the mean ± standard deviation (SD) of three independent experiments unless otherwise noted. One-way analysis of variance was used to investigate statistical significance by using SPSS (SPSS 21.0, Chicago, IL, USA, p-value < 0.05) with a post hoc test (Tukey).

## Results

### Characterization of CNT-PMMA

**Figure 1:**
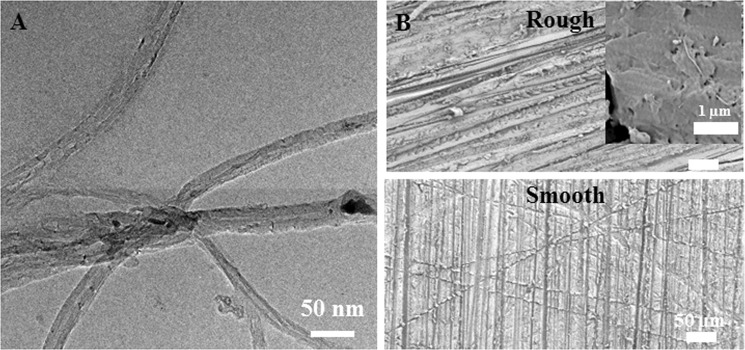
Morphology of CNTs and CNT-incorporated PMMA with rough or smooth surfaces. TEM images of (**A**) CNTs and representative SEM images of CNT-PMMA composites with rough or smooth surfaces. The insert in (**B**) is a highly magnified image showing CNTs (linear white material) on the surface.

**Figure 2:**
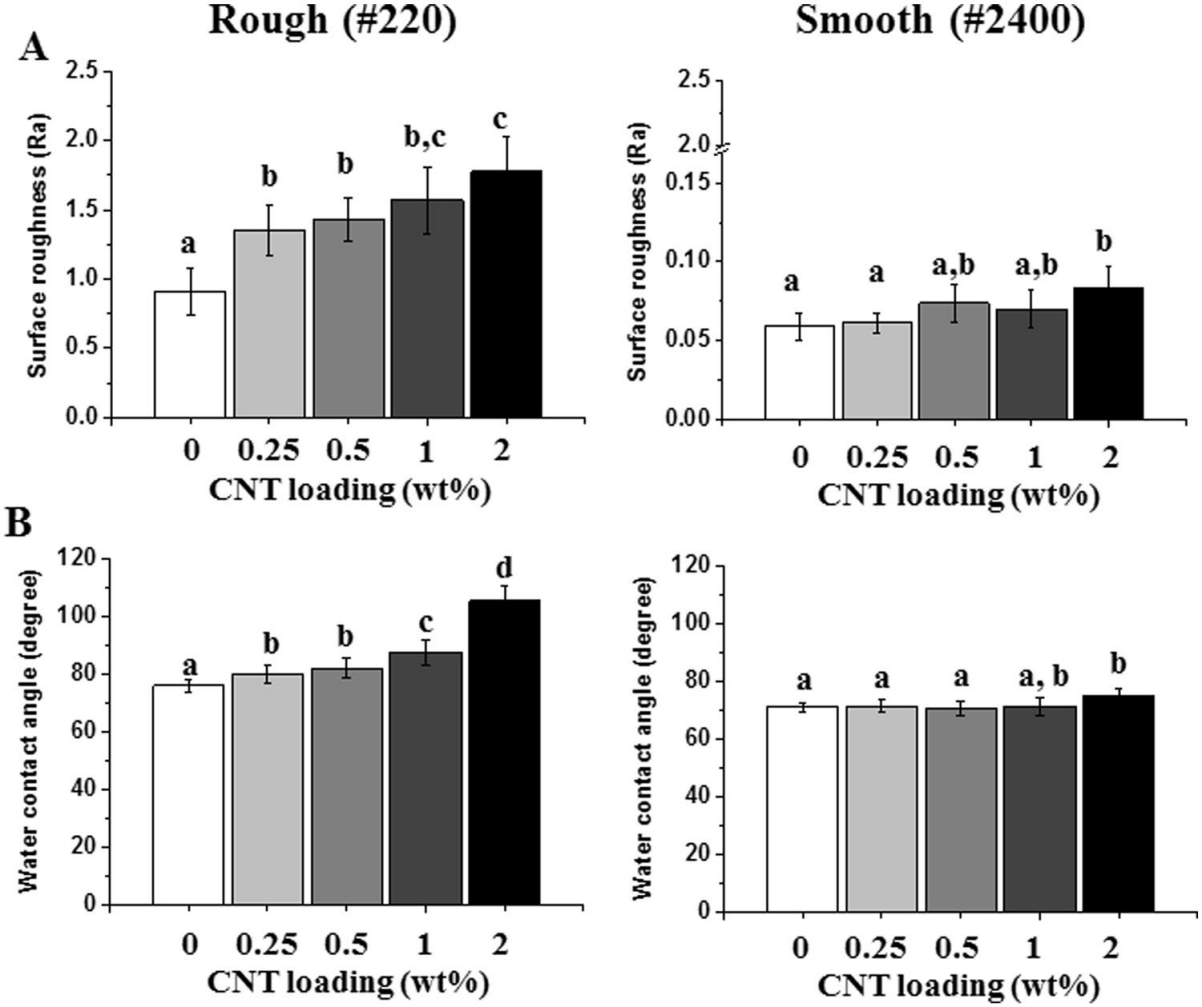
Increases in (**A**) surface roughness and (**B**) water contact angle after incorporating CNTs into PMMA. Increasing trends in roughness and water contact angles were observed for both rough and smooth surfaces. Different letters indicate significant differences among groups (n = 10 for (**A**) and n = 5 for (**B**), P < 0.05).

**Figure 3:**
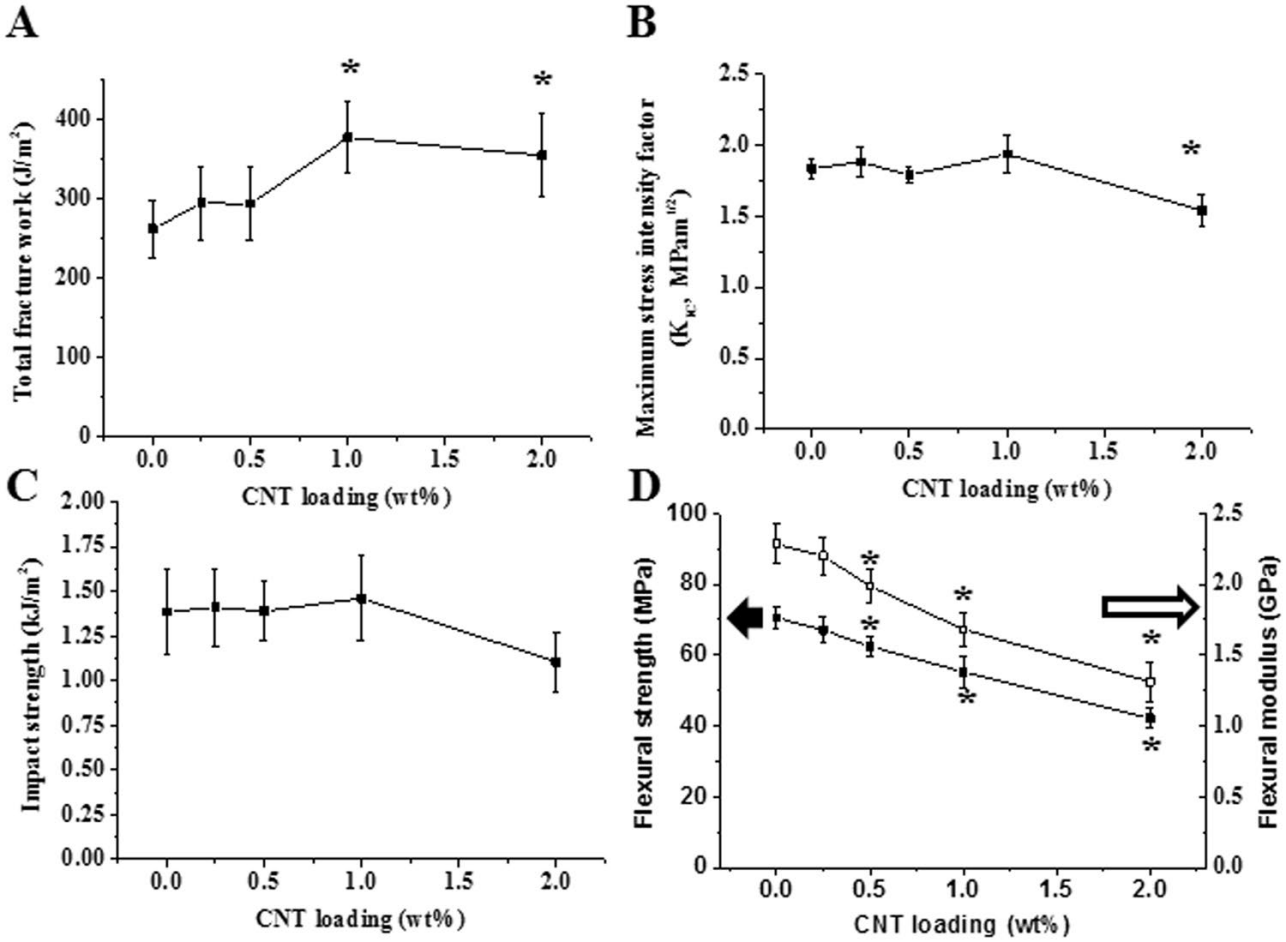
Mechanical properties of CNT-PMMA composites. (**A**) Total fracture work and (**B**) maximum stress intensity factor from fracture toughness test, (**C**) impact strength from Charpy impact test and (**D**) flexural strength and modulus from three-point flexural test. Enhanced total fracture work was observed in 1% and 2% CNTs. Asterisks (*) indicating statistical difference compared to 0% CNT (PMMA only) (n = 8, P < 0.05).

The morphological characteristics of carboxylated CNTs were revealed as multiwalled CNTs with an ~20 nm outer diameter (Fig. 1A). As a representative clinically applicable biomaterial used as a denture base resin, which is an oral mucosa replacement, PMMA was selected due to its simple fabrication method and its status as the environment most in contact with microbes in clinical settings. After carboxylation (-COOH) of CNTs, C=O and C-O peaks from carboxyl groups were clearly detected by FTIR (s Figure 1). The Turbiscan results showed sustained transmission (7~10% change) over time until 40 minutes, which confirmed the stability of carboxylated CNTs suspended in liquid MMA monomer (s Figure 2). After incorporating various amounts (up to 2%) of carboxylated CNT into PMMA, FTIR analysis showed elevation in the C=O, OH, and C-O peaks of CNTs without any peak shift (sFigure 1, 1724, 1401, and 1224 cm^−1^, respectively), depending on the content of carboxylated CNTs in the PMMA (s Figure 1)^32,50^. The FTIR results indicated physical bonding between the carboxylated CNTs and PMMA. SEM images of CNT-PMMA (Fig. 1B and s Figure 3) showed a severely roughened surface in specimens polished with 220 grit SiC paper (rough) compared to the surface of specimens polished with 2400 grit paper (smooth) in all groups. Highly magnified SEM images revealed long tube-like particles (white thin material) on the specimen surface, which were not detected on PMMA without added CNTs (Fig. 1B). The surface roughness tests showed significant increases in Ra for rough (220 grit) specimens relative to values for smooth (2400 grit) specimens, and increasing trends in roughness were observed in correlation with increases in CNT incorporation (Fig. 2A). For rough specimens, contact angle tests with DW showed a noticeable increase of the water contact angle in specimens with 1 and 2 wt% CNTs compared to that of the 0% CNT control specimens (p < 0.05, Fig. 2B). In the case of the specimens with a smooth surface, only those containing 2 wt% CNTs had a significantly higher water contact angle than the 0% CNT specimens.

### Mechanical properties

Significant increases in total fracture work (~40%) were observed in the 1 (P = 0.02) and 2 wt% (P = 0.03) CNT groups compared to the 0% CNT control group (Fig. 3A, P < 0.05), while the maximum stress intensity factor and impact strength were sustained at up to 1 wt% CNT incorporation (Fig. 3B, P > 0.05). The only significant decrease in maximum stress intensity compared to the 0% control group was observed at 2 wt% incorporation (*P* = 0.04, Fig. 3B, P < 0.05). In addition, significant decreases in flexural strength and flexural modulus, as gained by the 3-point flexural test results, were observed in all CNT groups except 0.25% CNT compared to the 0% control group (Fig. 3D, *P* < 0.05).

### Microbial adhesion-preventing effects

**Figure 4:**
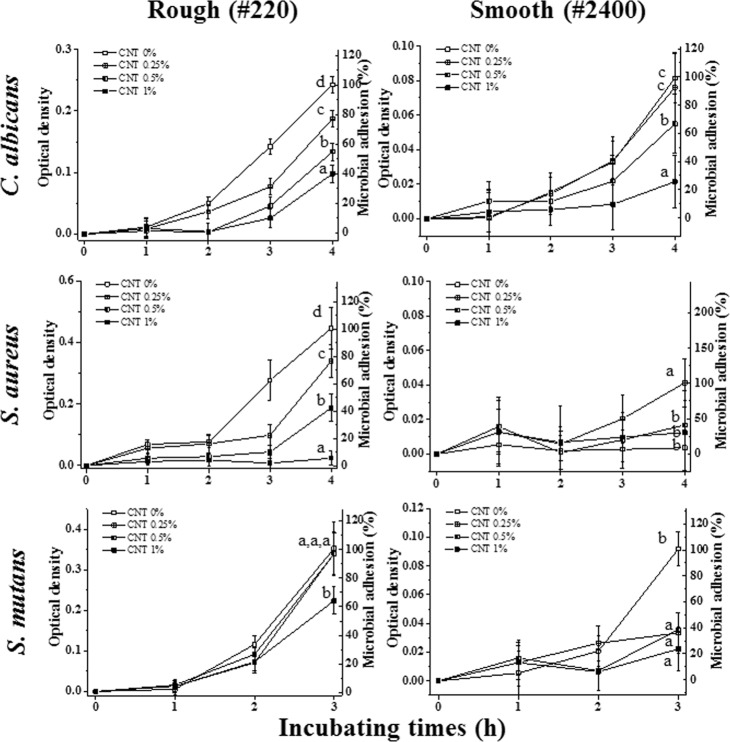
Anti-adhesive effects of CNT-incorporated PMMA against three different microorganisms. After microbial species (*C. albicans*, *S. aureus*, and *S. mutans*) were seeded on PMMA or CNT-PMMA for 1 h in artificial saliva, adherent microbes were cultured for specific incubation times (3–4 h) in microbial media. The attachment level of each species was determined by a PrestoBlue assay and normalized to that of CNT 0% (PMMA). Different letters indicate significant differences among them (n = 5, P < 0.05).

Based on the previous mentioned mechanical properties, the 2% CNT group was excluded from further experiments, as compared to the control group, it showed compromised mechanical properties in terms of flexural strength (and modulus), maximum stress intensity factor, and impact strength. Thus, the other groups (0.25, 0.5, and 1%) were chosen to investigate their immediate microbial adhesion-preventing effects. Overall, the anti-adhesion related antimicrobial ability against three microorganisms (*C. albicans, S. aureus*, and *S. mutans)* in artificial saliva was better on smooth surfaces than on rough surfaces (Fig. 4). Specifically, compared to the PMMA control group (0% CNT), the CNT-PMMA groups tended to show significant decreases in microbial adhesion (i.e., 35~95% less adhesion in the 1% CNT group) by all three microbial species for the rough surfaces, meaning that lower CNT incorporation resulted in greater microbial adhesion, i.e., 0% CNT ≥ 0.25% CNT ≥ 0.5% CNT ≥ 1% CNT in terms of microbial adhesion. Along with the abovementioned results, a similar anti-adhesion trend was detected on highly polished (smooth) surfaces, revealing that less CNT incorporation resulted in greater microbial adhesion. In particular, significantly decreased microbial adhesion (i.e., 60~85% less adhesion in the 1% CNT group) was observed in CNT-incorporated PMMA specimens compared to the specimens with 0% CNTs for *C. albicans* (0.5% and 1%), *S. aureus and S. mutans* (0.25%, 0.5%, and 1%) (Fig. 4. P < 0.05).

**Figure 5:**
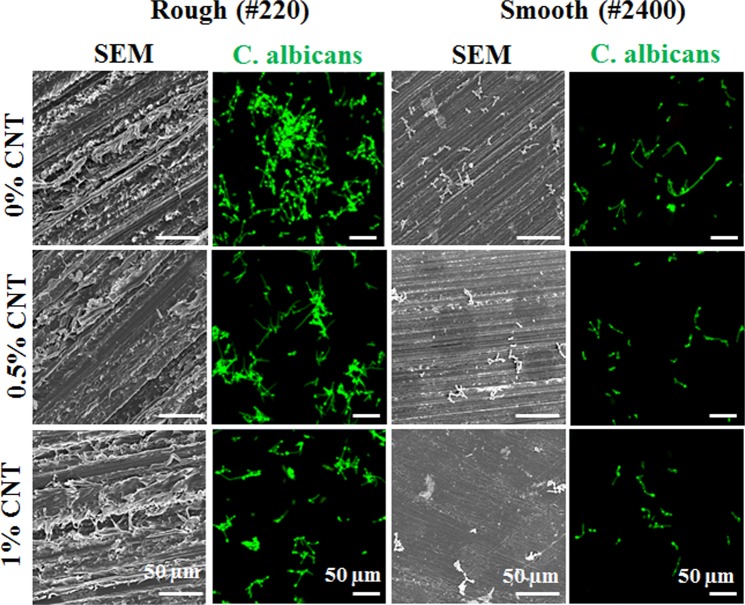
Anti-adhesive effects of CNT-incorporated PMMA against *C. albicans*. SEM images and images of adherent C. albicans were taken of a specimen. Fewer adherent cells were observed by SEM and confocal microscopy (green), respectively. Adherent cells on rough surfaces cannot be recognized visually without staining. Representative images were recorded after independent experiments were performed in triplicate.

Because of its major role in denture-associated oral infections, *C. albicans* was chosen to observe the anti-adhesive effects of CNT incorporation. The 0.25% CNT specimens were excluded from further study, as they were less effective than the other CNT-incorporated specimens in preventing microbial adhesion by *C. albicans*. Figure 5 shows SEM and fluorescence microscopy images. First, the SEM images of smooth surfaces showed that fewer *C. albicans* cells were present on CNT-incorporated specimens (0.5% and 1% CNTs) than on 0% CNT specimens; however, it was difficult to distinguish *C. albicans* on the specimens with rough surfaces due to the presence of PMMA debris on these surfaces. Therefore, the *C. albicans* cells were stained with FUN-1 dye, which clearly showed fewer *C. albicans* cells attached onto CNT-incorporated specimens (0.5% and 1% CNTs) than onto 0% CNT specimens.

**Figure 6:**
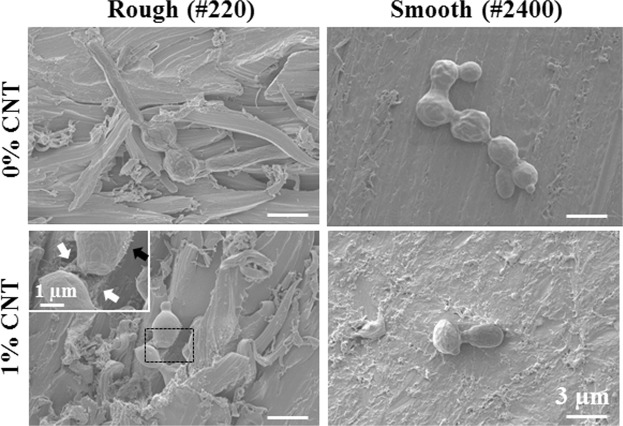
Disconnection of spores and lack of interaction between *C. albicans* and CNTs on the surface of specimens. Field emission SEM to obtain highly magnified images was utilized to observe interactions between *C. albicans* and CNT in detail on the surface of specimens. Autoaggregation of *C. albicans* consisting of more than 4 spores (4~7) were observed for rough and smooth surfaces with 0% CNT within 1 h of attachment, while single spores or two-spore aggregates were detected on surfaces with 1% CNT. Insert is a magnification of the black rectangle for 1% CNT on a rough surface, showing the disconnection of the interconnected filament of spores on the outer surface (black arrow) on an exposed CNT (white arrow) under the spores.

To observe the interaction between *C. albicans* and CNT-incorporated PMMA in greater detail, field-emission SEM was utilized to generate highly magnified images. Figure 6 shows autoaggregation of *C. albicans* involving more than 4 spores (4~7) on 0% CNT specimens with rough and smooth surfaces within 1 h of attachment, while only single spores or aggregates composed of two spores were observed on 1% CNT specimens. The insert (black rectangle) for a 1% CNT specimen with a rough surface shows disconnected spores with filaments on the outer surface (black arrow) not connected to the exposed CNT (white arrow) under the spore.

### Cytocompatibility testing against oral keratinocytes

**Figure 7:**
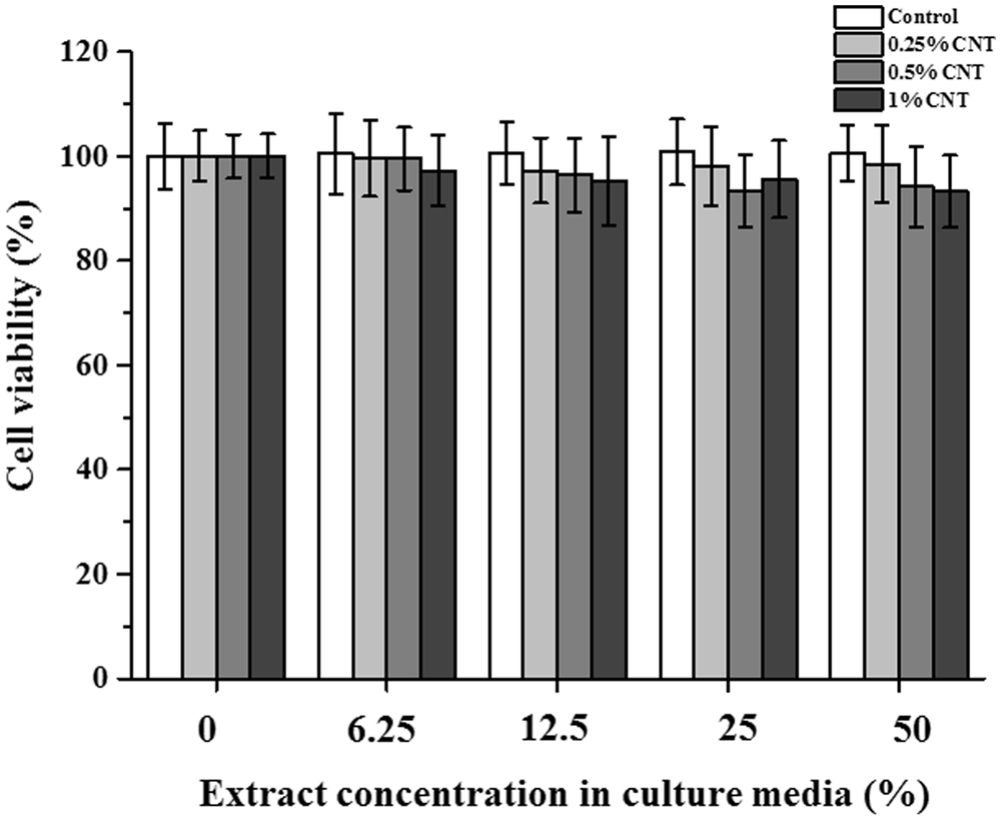
Cytocompatibility of CNT-incorporated PMMA with immortalized human oral keratinocytes (IHOKs). Adherent IHOKs were cultured with extracts for 24 h, and cell viability was analyzed by WST assay (n = 6).

Comparing cell viability in the control media revealed that extracts (50, 25, 12.5, and 6.25%) from CNT-PMMA specimens (0, 0.25, 0.5, and 1% CNTs) did not significantly diminish the viability of oral keratinocytes (Fig. 7, *P* > 0.05), revealing the cytocompatibility of CNT-incorporated PMMA with the oral keratinocytes present in the outer layer of the oral mucosa.

## Discussion

This current study utilized carboxylated CNTs as an intriguing additive in PMMA to produce anti-adhesive properties via the intrinsic antimicrobial abilities of CNTs. We revealed that incorporating carboxylated CNTs into PMMA alleviated the intensities of FTIR peaks regarding carbonyl, hydroxyl, and ether groups, which are peaks that are characteristic of carboxylated CNTs. After confirming the suspension stability of the carboxylated CNTs in the monomer solution, the CNT-monomer liquid complex was physically mixed with PMMA powder. Thus, CNT incorporation can be expected to enhance myriad aspects of PMMA. We created rough and smooth surfaces using different polishing treatments to mimic clinically relevant situations of denture use: scratched denture surfaces while in use and freshly polished surfaces when delivered to patients. SEM analysis displayed that CNTs were exposed on the surface, especially on the roughened 2% CNT specimen, whereas the CNTs were not easily visualized by SEM in the other specimens. The FTIR results showed that the intensities of peaks related to carboxylated CNTs increased as the amounts of CNTs incorporated increased, indicating successful incorporation of the carboxylated CNTs into PMMA and indirectly revealing the surface exposure of the CNTs. However, homogeneous CNT incorporation into the PMMA matrix was not able to be successfully performed using the current CNT-PMMA system. If CNTs were homogeneously dispersed in the matrix and controllably exposed outwardly, the characteristics of the composite, such as the physico/mechanical and anti-microbial adhesion properties, would be further enhanced. Therefore, the development of methods for homogeneously incorporating CNTs in the resin matrix should be further investigated using other CNT functionalization conditions, such as PEG, salinization, SDS surfactant and palmitic acid^51,52^. Within the limitations of this CNT-PMMA composite morphology, the possibly exposed CNTs can increase both the surface roughness and the water contact angle, as shown by the current cauterization of the composite.

Before investigating the anti-adhesive effects of the composite, its mechanical properties related to fracture toughness, including total fracture work, maximum stress intensity factor, impact strength, and 3-point flexural strength/modulus, were analyzed. The mechanical properties are not the key property for endowing PMMA denture resin with anti-adhesive effects. However, dentures that are easy to fracture cannot be used for clinical applications. Thus, the mechanical properties were also investigated for optimizing the specimen conditions, meeting the mechanical requirements of the ISO standard for dentures and maintaining mechanical parameters that are comparable to the commercially available control (0% CNTs). The total fracture work, indicating the resistance to external deforming energy until fracture, was significantly increased for the 1% and 2% CNT groups compared to the 0% CNT control group, while compared to that of the 0% CNT group, the maximum stress intensity factor, another parameter of fracture toughness describing the maximum stress until fracture, of the 0.25, 0.5 and 1% CNT groups was maintained. By contrast, the maximum stress intensity factor of the 2% CNT group was compromised. Similar inconsistent results regarding the parameters from fracture toughness were similarly observed in previous efforts to incorporate CNT in PMMA^53^. Impact strength, indicating resistance to a sudden extrinsic force in the case of an unexpected drop of dentures or a force applied to dentures while in use, was also significantly compromised at 2% CNT incorporation. Unfortunately, the impact strength of all types of specimens failed to meet the minimum requirements, which were suggested by the previous ISO standard (1567) for only specific products with enhanced mechanical properties and were later replaced by fracture toughness in the revised ISO standard (20795-2). In this study, impact strength was only used for comparing experimental dentures. Because of the lower values of flexural strength/stiffness of the 2% CNT specimens than the ISO standard (50 and 1500 MPa, respectively) for chemically activated denture resin, the 2% CNT group was excluded from further tests of antibacterial effects and cytocompatibility. The standard value of each mechanical parameter is summarized in Table 1, with a statistical increase/decrease of each value from the CNT groups compared to the control group. Other investigators have also reported that up to 0.5~1% CNT incorporation into biopolymers (i.e., PMMA polyester, polystyrene, nylon-6) did not impair mechanical properties, whereas CNTs at high loadings relative to PMMA (over 2%) might act to weaken the microstructure of the composite under the stress of extrinsic forces, especially when they are aggregated in the matrix^53–55^.

**Table 1:** Mechanical properties and the minimum value of PMMA-based denture resin according to the ISO standard (20795-2(a) and 1567(b)).

	Fracture toughness		Impact strength^b^	3 point flexural test		Biological test
	Total fracture work^a^	Maximum stress intensity factor^a^		Ultimate flexural strength^a^	Elastic modulus^a^	
Standard	250 J/m^2^	1.1 Mpam^1/2^	2 kJ/m^2^	50 MPa	1500 MPa	Included
0% CNT	+	+	−	+	+	Included
0.25% CNT	+	+	−	+	+	Included
0.5% CNT	+	+	−	+ down	+ down	Included
1% CNT	+ up	+	−	+ down	+ down	Included
2% CNT	+ up	+ down	− down	− down	− down	Excluded

+/− indicating whether the average value of each group met the required value from the standard or not.

Up/down indicates significant increase or decrease in average value of each group compared to the 0% control group at a level of 0.05.

Three different representative microorganisms (*C. albicans, S. mutans* and *S. aureus*) were selected for characterizing the anti-adhesive effects of CNT-PMMA composites because of their major infection roles in oral micro environment during dental practice, and artificial saliva was used as the basal media in adhesion tests to mimic the oral microenvironment^41^. CNT-PMMA exhibited anti-adhesive effects against all investigated microorganisms, and the anti-adhesive effects were enhanced with increasing CNT incorporation. In agreement with previous studies, increased roughness and decreased water affinity were considered to have correlation with increases in microbial adhesion on biomaterials including PMMA^56–58^. SEM images and florescent images of cells on the substrate were taken to confirm the above anti-adhesive properties of CNT-PMMA. The 0.25% CNT group was excluded because of the small difference from the control group in anti-adhesive properties due to the relatively small amounts of CNT. Among the three different microorganisms tested, *C. albicans* was chosen as the representative microbial species related to the most common denture-derived oral infection (i.e., Candidiasis). On rough surfaces, a more significant reduction in *C. albicans* adhesion was observed for 1% CNT specimens (up to ~60%) compared to 0% CNT specimens; on smooth surfaces, all specimen showed much reduced microbial adhesion (60~80%) compared to that on rough surface and CNT incorporated specimens showed more adhesion reductions up to 70% over increase of CTN amount. In agreement with previous studies, rough PMMA surfaces are more vulnerable to microbial species adhesion than smooth surfaces. When CNTs are incorporated in PMMA and the surface is scratched or roughened by clinical usage (or experimentally polished by SiC paper), more CNTs are exposed on the outer surface due to the increased surface area relative to that of a smooth surface. This conclusion was confirmed by the almost twenty-fold increase in surface roughness: for a rough surface, 1% CNT specimens showed an ~1.6 µm Ra (vs ~0.8 µm Ra for 0% CNT), whereas for a smooth surface, 1% CNT specimens showed an ~0.08 µm Ra (vs ~0.06 µm Ra for 0% CNT). In addition to the high degree of adhesion reduction against *C. albicans*, reductions of ~95% and ~30% against adhesion by *S. aureus* and *S. mutans*, respectively, were also observed. These effects of CNTs are in line with the results from other studies demonstrating the anti-adhesion effects of CNTs against microbial species^59–61^. To date, this study is the first to reveal anti-adhesion effects of CNT-biopolymer composites without any added antimicrobial drugs, biomolecules, or ions (e.g., Ag, Cu, Gd) against microbes.

Surfaces resistant to microbial adhesion are commonly achieved with PMMA by creating either zwitterionic or hydrophilic surfaces, which help generate a tightly bound water layer; this layer creates a physical/energetic barrier that prevents the adhesion of microbial species^62,63^. In contrast, when CNTs were incorporated into a composite with PMMA, an increase in hydrophilicity was not observed; instead, the hydrophilicity decreased, and the water contact angle increased. Therefore, the anti-adhesive properties of CNT-PMMA must be explained by other mechanisms. When microbes were directly exposed to CNTs, phospholipid extraction, membrane cutting, and the disconnection of sequential microbe chains (via interconnected filaments) were revealed^26,60,61^. According to the SEM images and the staining of adherent cells, the disconnection of spores was observed only in the absence of dead microbes on the surface, which indicates that the disconnection of sequential microbe chains plays a major role in the antimicrobial adhesion mechanism of CNTs. During washing to detach unattached microbes, unattached damaged or dead microbes were washed away, and adherent microbes remained.

Possible toxic response can occur when CNT-PMMA encounters the outermost layers of the oral tissue in clinics. Thus, cytotoxicity was investigated using oral keratinocytes as representatives of the cells in the outermost mucosal layers^64^. The cytotoxicity results indicate that compared to the control, none of the CNT-PMMA composites exerted adverse effects. To date, PMMA has not been reported to induce systemic toxic effects in humans after fully polymerized^8^. However, CNTs and their composites have not yet been approved for clinical usage by the FDA or equivalent organizations in any country. Thus, along with the cytocompatibility results against oral mucosa keratinocytes, future *in vitro* or *in vivo* studies regarding the use of CNT-PMMA are necessary to confirm the biocompatibility of these compounds and their suitability for clinical use.

## Conclusion

In the present study, we successfully developed CNT-PMMA with drug-free antimicrobial-adhesive properties to prevent microbe-induced complications. CNT-PMMA composites showed exposed CNTs on their surface, which increased the surface roughness and water contact angle. Among specimens with CNT incorporation ranging from 0.25~2%, 1% CNT was chosen as the optimal concentration for use as an additive to PMMA due to its sustained mechanical properties and striking reduction in microbial adhesion (30~95% adhesion reduction). Overall, this work provides information on the utilization of nanoparticles (e.g., CNTs) for the development of promising clinically applicable biomaterials (e.g., PMMA) with properties that prevent microbial adhesion. These materials could be used for removable or provisional dental biomaterials (i.e., base resin for dentures, orthodontic appliances, or provisional restoration) and implantable biomaterials.

## Supplementary information

Supplemental appendix
