# Supplementary material for: Carbon nanotube incorporation in PMMA to prevent microbial adhesion
Source: Sci Rep. 2019 Mar 20;9:4921. doi: 10.1038/s41598-019-41381-0 (PMC6427005; doi:10.1038/s41598-019-41381-0)
Supplement: Supplementary file 1 — Supplemental appendix [file 41598_2019_41381_MOESM1_ESM.doc]

**Supplemental appendix**

**Carbon nanotube incorporation in PMMA**

**to prevent microbial adhesion**

Kyoung-Im Kim^1,*^, Dong-Ae Kim^2,3,*^, Kapil D. Patel^1,2,4,5^, Ueon Sang Shin^2,4^, Hae-Won Kim^1,2,4,5^, Jung-Hwan Lee^1,2,5,#^, Hae-Hyoung Lee^1,2,5,#^

^1^Department of Biomaterials Science, School of Dentistry, Dankook University, Cheonan 31116, South Korea

^2^Institute of Tissue Regeneration Engineering (ITREN), Dankook University, Cheonan 31116, South Korea

^3^Department of Dental Hygiene, Kyungwoon University, Gumi-si, South Korea

^4^Department of Nanobiomedical Science & BK21 PLUS NBM Global Research Center for Regenerative Medicine Research Center, Dankook University, Cheonan 31116, South Korea

^5^UCL Eastman-Korea Dental Medicine Innovation Centre, Dankook University, Cheonan 31116, Republic of Korea

**#Co-Corresponding Authors**

Jung-Hwan Lee, DDS, PhD, Researcher, Institute of Tissue Regeneration Engineering (ITREN), Dankook University, Cheonan 31116, Republic of Korea,

Tel) +82 41 550 3081; Fax) +82 41 559 7839; E-mail) ducious@gmail.com

Hae-Hyoung Lee, DDS, PhD, Professor, Department of Biomaterials Science, College of Dentistry, Dankook University, Cheonan 31116, Republic of Korea

Tel) +82 41 550 3081; Fax) +82 41 550 3085; E-mail) [haelee@dku.edu](mailto:haelee@dku.edu)

* Equally contributed as first authors

**
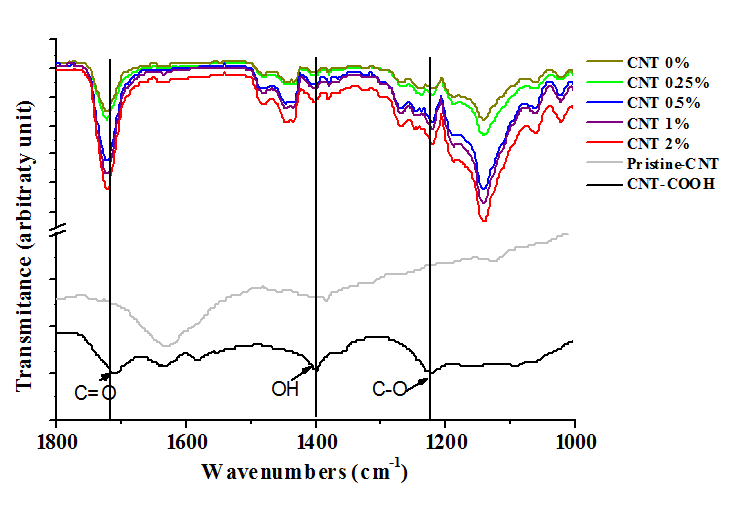
**

**sFigure 1 Characteristics of CNTs, carboxylated CNTs and carboxylated CNT-incorporated PMMA.** FTIR peaks from carboxylated CNTs in composites increased with increasing carboxylated CNT concentrations up to 2 wt%.

**
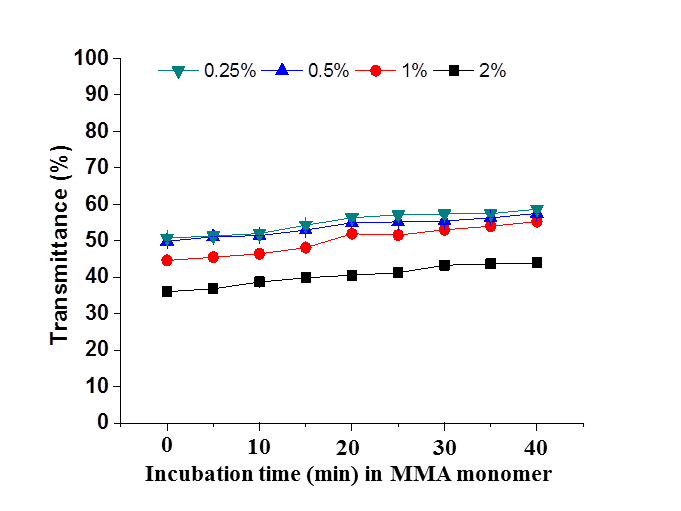
**

**sFigure 2. CNT dispersion stability in liquid MMA monomer measured by Turbiscan.** The Turbiscan results showed sustained transmission (7~10% change) over time until 40 minutes, which confirmed the stability of carboxylated CNTs suspended in liquid MMA monomer.


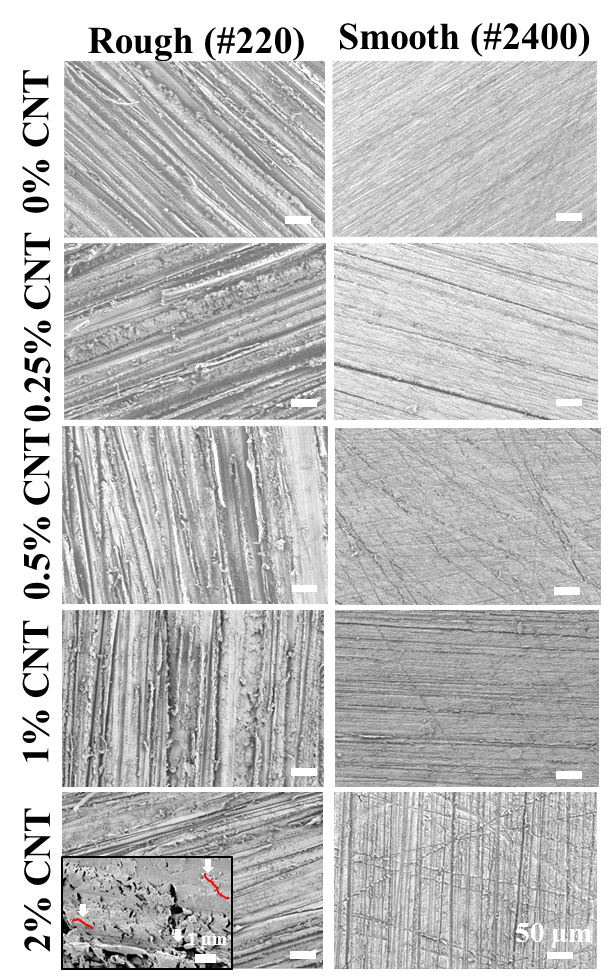


**sFigure 3. Surface morphology of CNT-incorporated PMMA with rough and smooth surfaces created by polishing with papers of different grit sizes (#220 *vs* up to #2400), visualized by SEM**. The insert for 2% CNT with a rough surface is a highly magnified SEM image confirming the morphological presence of CNTs in PMMA (pseudo red colored, indicated by white arrows).
